# Supplementing Dairy Ewes Grazing Low Quality Pastures with Plant-Derived and Rumen-Protected Oils Containing Eicosapentaenoic Acid and Docosahexaenoic Acid Pellets Increases Body Condition Score and Milk, Fat, and Protein Yields

**DOI:** 10.3390/ani8120241

**Published:** 2018-12-19

**Authors:** Quang V. Nguyen, Hung V. Le, Don V. Nguyen, Peter Nish, John R. Otto, Bunmi S. Malau-Aduli, Peter D. Nichols, Aduli E. O. Malau-Aduli

**Affiliations:** 1 Animal Genetics and Nutrition, Veterinary Sciences Discipline, College of Public Health, Medical and Veterinary Sciences, Division of Tropical Health and Medicine, James Cook University, Townsville, Queensland 4811, Australia; quang.nguyen2@my.jcu.edu.au (Q.V.N.); vanhung.le@my.jcu.edu.au (H.V.L.); donviet.nguyen@my.jcu.edu.au (D.V.N.); john.otto@jcu.edu.au (J.R.O.); Peter.Nichols@csiro.au (P.D.N.); 2 College of Economics and Techniques, Thai Nguyen University, Thai Nguyen 252166, Vietnam; 3 National Institute of Animal Science, Thuy Phuong, Bac Tu Liem, Hanoi 129909, Vietnam; 4 TasHerd Pty Limited, P.O. Box 68, Hadspen TAS 7290, Australia; peter.nish@tasherd.com.au; 5 College of Medicine and Dentistry, Division of Tropical Health and Medicine, James Cook University, Townsville, Queensland 4811, Australia; bunmi.malauaduli@jcu.edu.au; 6 CSIRO Oceans & Atmosphere, P.O. Box 1538, Hobart TAS 7001, Australia

**Keywords:** PUFA, oils, body condition score, sheep milk composition, supplementation, canola, flaxseed, safflower, rice bran

## Abstract

**Simple Summary:**

This study evaluated the lactation performance and body condition scores of purebred Awassi and Awassi × East Friesian crossbred dairy ewes grazing low quality pastures and supplemented with diverse plant-derived oil enriched pellets under on-farm management conditions. The origin and treatment of eicosapentaenoic acid (EPA) and docosahexaenoic acid (DHA) to get the rumen protected EPA + DHA treatment was based on a modification of the microencapsulation of oil droplets in a protein-aldehyde matrix procedure. The results demonstrated that supplementation with rumen (EPA + DHA) and oil-infused pellets improved milk, fat, and protein yields by approximately 30%, 13%, and 31% respectively, and crossbred ewes produced more milk than purebreds. These results are very useful for dairy sheep producers in improving ewe lactation performance, milk quality, and body condition score under low quality pasture grazing conditions.

**Abstract:**

The Australian dairy sheep industry is small and mostly based on a natural grass grazing system, which can limit productivity. The current study tested different plant oil-infused and rumen protected polyunsaturated fats and their interactions with sire breeds to improve lactation traits and body condition scores (BCS) of ewes grazing low quality pastures. It was hypothesised that supplementing lactating ewe’s diets with plant-derived polyunsaturated oils would improve milk production and composition without compromising BCS. Sixty ewes (*n* = 10/treatment) in mid-lactation, balanced by sire breed, parity, milk yield, body condition score, and liveweight, were supplemented with: (1) control: wheat-based pellets without oil inclusion; wheat-based pellets including; (2) canola oil (CO); (3) rice bran oil (RBO); (4) flaxseed oil (FSO); (5); safflower oil (SFO); and (6) rumen protected marine oil containing eicosapentaenoic acid and docosahexaenoic acid (RPO). Except for the control group, all supplementary diets included the same level of 50 mL/kg DM of oil and all diets were isocaloric and isonitrogenous. Experimental animals were grazed in the same paddock with *ad libitum* access to pasture, hay, and water during the 10-week study. RPO was the most effective diet that enhanced milk, fat, and protein yields by approximately 30%, 13%, and 31%, respectively (*p* < 0.0001). A significant increase in milk production was also observed with CO, RBO, and SFO treatments (*p* < 0.0001). Breed significantly influenced animal performance with higher milk yields recorded for crossbred Awassi × East Friesian (AW × EF) (578 g/day) vs. purebred Awassi (452 g/day) (*p* < 0.0001). This study provides empirical evidence for the use of rumen-protected and plant-derived oil-infused pellets as supplements under low quality pasture grazing conditions to improve the production performance of purebred Awassi and crossbred AW × EF ewes.

## 1. Introduction

Although previously published studies have demonstrated that sheep milk has more nutritional value compared to cow milk [1,2], the contribution of milk derived from sheep to national milk production in Australia is relatively low. As of 2013, there were 13 commercial farms producing 550,000 litres of milk annually [3] compared to 9 billion litres of milk produced by dairy cows nationwide [4]. Milk yield and composition are influenced by various factors, including diet, breed, age, management practices, health, and the environment [5,6,7]. Dietary supplementation with fat is considered as an effective tool to improve milk yield and alter milk composition [8,9]. Plant derived oils are a potential source of dietary fat and have been used in ruminant feeds to increase the energy density of diets and modify the milk fatty acid profile [7,10,11], with the aim of increasing n-3 long–chain (≥C20) polyunsaturated fatty acids (n-3 LC-PUFA) in dairy products. This is because high consumption of n-3 LC-PUFA in humans has been demonstrated to inhibit adipogenic, diabetogenic, atherogenic [12], inflammatory [13,14], and carcinogenic [15] diseases and lower the risk of developing Alzheimer’s disease [16]. A number of authors have demonstrated that while dietary fat supplements can enhance milk yield [17,18,19,20], it is accompanied by a decrease in milk fat and protein composition because of the negative correlation between milk solid concentration and milk yield [7,21]. This could reduce the income of the producers as milk is generally traded based on total milk solids. For this reason, the use of fats as dietary sources to improve the milk yield of sheep used for commercial milk harvesting within Australia is not widely undertaken and is mostly applied as a supplement only during the dry seasons when pasture quality and quantity are low, in order to increase the energy intake of lactating animals [22]. 

To our current knowledge, studies on the effect of dietary supplementation with rice bran, canola, and safflower oils on milk yield and composition have only been conducted with dairy cows [19,23,24] and goats [25], but not dairy ewes. The effects of supplementation with flaxseed on animal performance and milk fatty acid profiles have been studied with dairy ewes, however, these investigations supplemented flaxseed either as whole or extruded grain [26,27,28]. In addition, there has been a paucity of studies that have examined the effects of varying dietary supplementation on lactation and liveweight traits in grazing dairy ewes of different genetic backgrounds under the same management and feeding regime. 

The major objective of the current work was to fill these knowledge gaps by comparing the lactation performance, milk composition, and body condition score of dairy ewes in mid lactation grazing low quality pastures and supplemented with canola, rice bran, flaxseed, safflower, and rumen protected oil-infused pellets. It was hypothesised that supplementing grazing dairy ewes with oils of different plant-derived and marine origins will have different effects on milk yield, milk composition, and body condition score.

## 2. Materials and Methods

### 2.1. Animal Ethics

The use of animals and procedures performed in this study were all approved by the University of Tasmania Animal Ethics Committee (Permit No A0015657).

### 2.2. Animal Management and Experimental Design

Sixty lactating Awassi and crossbred Awassi × East Friesian ewes in mid-lactation, located in the South East of Tasmania (Grandvewe Cheeses Farm, Birchs Bay, Woodbridge, Tasmania, Australia), were included in a 10-week feeding trial where the ewes were kept in the same paddock and had *ad libitum* access to local natural velvet tussock grass, hay, and water. The experimental animals were allocated to six dietary treatments with each group balanced for liveweight, breed, parity, body condition score (BCS), and milk yield. Treatments consisted of (1) commercial wheat-based pellets without oil inclusion (control); wheat-based pellets infused with 50 mL/kg DM of (2) canola (CO); (3) rice bran (RBO); (4) flaxseed (FSO); (5) safflower (SFO), and (6) rumen protected EPA + DHA (RPO) oils, as represented in Table 1. All treatments were isocaloric and isonitrogenous (Table 2). Each ewe was fed 1 kg/day of the supplemented pellet individually in the milking parlour during milking time over a 10-week period with an initial two-week adjustment period, followed by an 8-week experimental period. In the first two weeks of the adjustment period, commercial pellets (control) for each treatment group were increasingly substituted at 100 g/day by the experimental diets, CO, RBO, FSO, SFO, and RPO, until the attainment of 1 kg/day on day 10 was achieved. Ewes were milked in the mornings at 0600 h and individual milk yield was electronically recorded by the La Laval platform using De Laval’s Alpro Herd Management System software version 6.54 (De Laval, Tumba, Sweden).

### 2.3. Feed Intake and Body Condition Score

The amount of offered pellets and residuals were weighed daily to calculate feed intake. Weekly feed samples were collected and stored at −20 °C for subsequent chemical analysis. Body condition score (BCS) was subjectively evaluated at weekly intervals on a scale of 1–5 [29] by the same evaluator to ensure consistency and repeatability. 

### 2.4. Milk Sample Analyses

Weekly milk samples from each animal were bulked from daily milkings at 0600 h and stored in labelled plastic vials containing bromopol blue preservative at 4 °C before sending the samples off to Hadspen for compositional analysis at the officially contracted herd recording laboratory–TasHerd Pty Ltd., (Hadspen, Tasmania, Australia). Fourier transformed infrared spectrometry (Bentley Fourier Transform Spectrometer, Chaska, MN, USA) was used to quantify milk composition. This system uses Bentley Flow Cytometry to measure the somatic cell count, while the Bentley Fourier Transform Spectrometer measures somatic cell count, milk fat, protein, and lactose based on an official laboratory analysis method [30]. The equation from Mavrogenis and Papachristoforou [31] was used to calculate the fat-corrected milk (FCM):

6% FCM = M (0.453 + 0.091F)

where “F” is the percentage of fat and “M” is the milk yield (kg).

### 2.5. Chemical Analysis of Experimental and Basal Diets

Before analysing dry matter (DM), ash, and chemical composition, samples of the basal and experimental diets were dried in a fan-forced oven at a constant temperature of 65 °C and subsequently ground through a 1 mm sieve using a Thomas Model 4 Laboratory Mill (Thomas Scientific, Swedesboro, NJ, USA). DM content was determined by placing the ground samples at 150 °C in an oven for 24 h to remove moisture. The samples were combusted in a furnace set at 600 °C for 8 h to determine ash content. Neutral detergent fibre (NDF) and acid detergent fibre ADF were quantified using an ANKOM220 fibre analyser, while an ANKOM^XT15^ fat/oil extractor (ANKOM Technology Corp., Macedon, NY, USA) was used to measure ether extract. The crude protein percentage was calculated based on the value of nitrogen that was determined using a Thermo Finnigan EA 1112 Series Flash Elemental Analyser (Thermo Fisher Scientific, Waltham, MA, USA). Table 2 shows the nutritional composition of the experimental diets.

### 2.6. Data and Statistical Analysis

All data were analysed using ‘Statistical Analysis System’ software [32]. Initial descriptive summary statistics were computed with means, standard errors, and minimum and maximum values scrutinised for data entry errors and outliers. The data were then subjected to general linear model (PROC GLM) analysis, with different oil supplementation, sire breed, week of supplementation, and their interactions fitted as fixed effects and feed intake, milk yield, milk composition, and body condition score as dependent variables. The level of significance threshold was *p* < 0.05 and differences between means were established using Duncan’s multiple range and Turkey’s probability pairwise comparison tests. The final statistical model used for the analysis was:

Yijk = μ + SB_i_ + D_j_ + W_k_ + (SBD)_ij_ + (SBW)_ik_ + (DW)_jk_ + e_ijk_

where Y_ijk_ is the dependent variable, μ is the overall mean, SB, D, and W are the fixed effects of sire breed, diet, and week of supplementation, respectively, brackets represent second-order interactions, and e_ijk_ is the error term. 

## 3. Results

The results of this study suggest that dietary treatments significantly influenced feed intake of grazing dairy ewes (*p* < 0.0001; Table 3), with DM intakes being greatest in the control group, followed by the RBO, SFO, CO, RPO, and FSO groups, respectively. Estimated intake of OM, ADF, NDF, and CP followed a similar pattern to DMI, with the greatest intakes observed in the control group, except the intake of EE, which was greatest in the RBO group (41 g/day). Breed and its interaction with supplementation had no significant effect on intake (DMI), and was therefore excluded from Table 3.

Significant differences in dairy performance traits, milk composition, and body condition score were observed across treatments (Table 4). Ewes receiving RPO produced the greatest milk yield at 628 g/day, followed by SFO, RBO, CO, FSO, and the control (*p* < 0.0001). Inconsistent with milk yield, fat concentration was highest in milk from the control (*p* = 0.015), whereas RBO yielded the greatest content of protein (5.9 g/100 g) (*p* < 0.0001), resulting in the highest concentration of solids-non-fat (11.7 g/100 g) in this group. Although milk from ewes fed RPO had the least proportion of fat and protein at 6.6 and 5.4 (g/100 g), respectively, this group produced the greatest fat yield (FY) (41 g/day; *p* = 0.0008) and protein yield (34 g/day; *p* = 0.0004). There were no significant differences among treatments in the percentage of milk lactose. The type of oil included in the dietary supplement affected body conformation (*p* = 0.0008), although the mean BCS of experimental ewes only varied from 2.2–2.4 (Table 4). Since the cell counts for healthy sheep range from 10 to 200 × 1000 cells/mL, cell counts of all treatments ranged from 60 to 109 × 1000 cells/mL (Table 4), indicating that all experimental animals were free from intramammary infections during the feeding trial. 

It was evidenced that breed also had major impacts on milk production rather than milk composition (Table 4), with significantly higher milk (*p* < 0.0001), fat (*p* < 0.0001), and protein (*p* < 0.0001) yields observed in crossbred AW × EF than purebred AW. There were minor variations in terms of mean fat, protein, and lactose contents between AW × EF and AW, despite the statistical difference in lactose percentage. 

**Figure 1:**
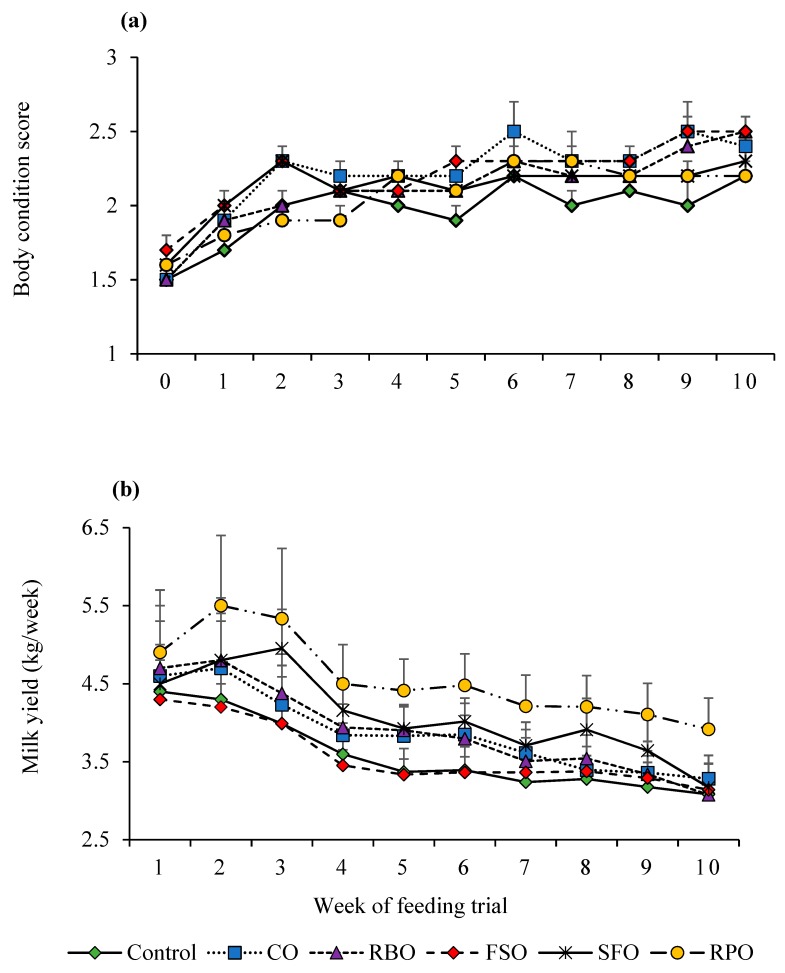
Weekly trends in body condition score (**a**) and milk yield (**b**). Canola oil (CO), rice bran oil (RBO), flaxseed oil (FSO), safflower oil (SFO), rumen-protected oil (RPO).

**Figure 2:**
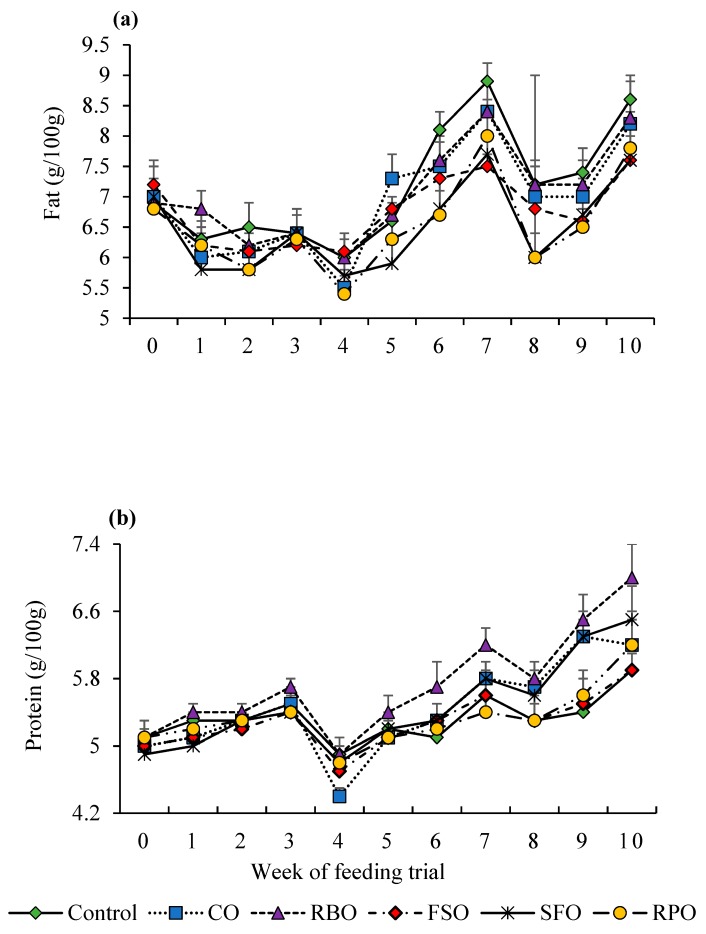
Weekly trends in milk fat (**a**) and milk protein (**b**) concentration. Canola oil (CO), rice bran oil (RBO), flaxseed oil (FSO), safflower oil (SFO), rumen-protected oil (RPO).

Weekly trends for BCS and lactation traits are presented in Figure 1 and Figure 2. As observed in all treatment groups, BCS, fat percentage, and protein percentage (Figure 1a and Figure 2a,b) increased, while milk yield decreased over the duration of the experimental period (Figure 1b). The best weekly milk yield trend was recorded in the RPO group, where its decrease was smaller (4.9 at the start to 3.9 kg/week) than the other groups at the end of the trial.

**Figure 3:**
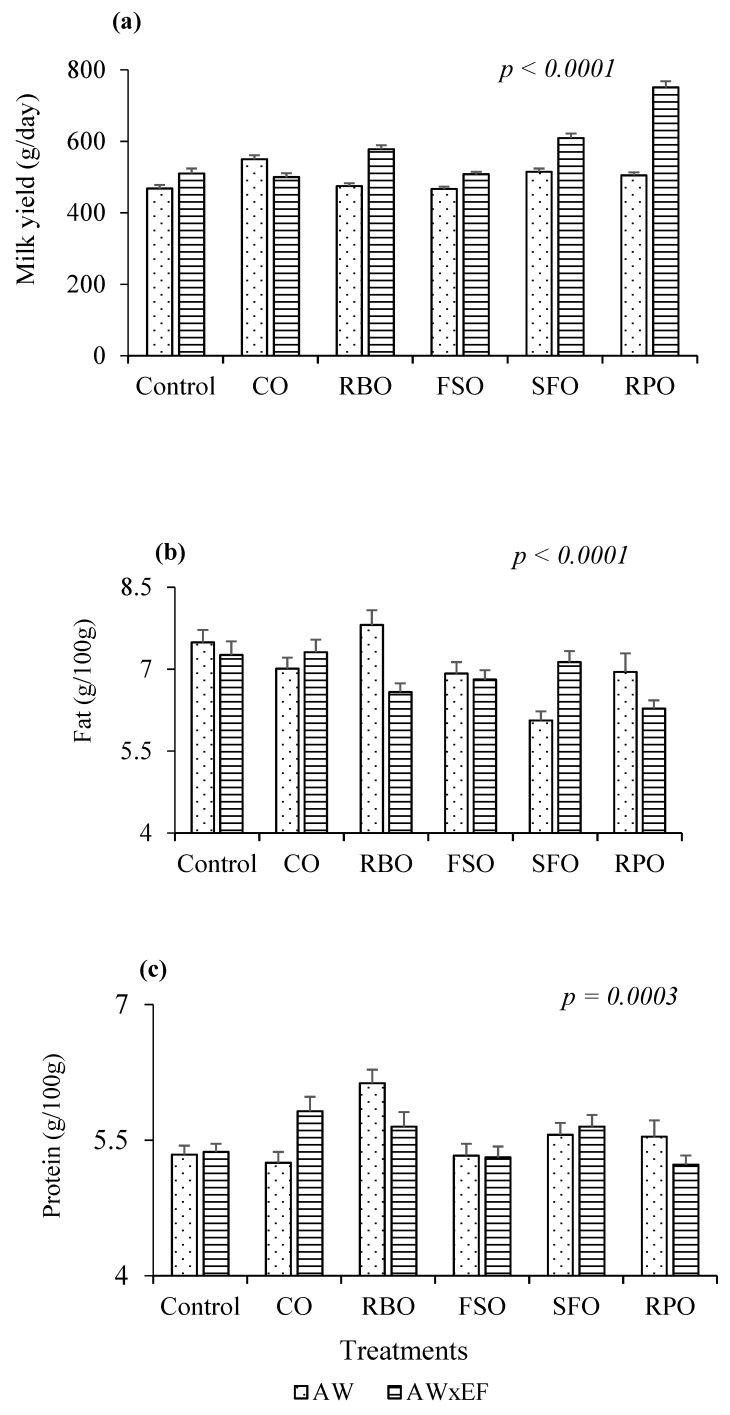
Supplementary diet and breed interactions on (**a**) milk yield; (**b**) milk fat; and (**c**) milk protein. Canola oil (CO), rice bran oil (RBO), flaxseed oil (FSO), safflower oil (SFO), rumen-protected oil (RPO).

Figure 3 presents significant interactions between oil supplementation and breed in milk yield (*p* < 0.0001), fat percentage (*p* < 0.0001), and protein percentage (*p* = 0.0003). Regarding milk production, crossbred AW × EF ewes had greater responses to oil supplements than AW with the highest milk yield at 751 g/day observed in the RPO group (Figure 3a). Breed and diet interactions, however, were varied across treatments in which AW ewes fed with RBO produced the highest percentages of fat and protein (7.8 and 6.1 g/100 g, respectively).

## 4. Discussion

### 4.1. Effect of Dietary Supplements on Dry Matter Intake and Body Condition Score

The decrease in DMI was inconsistent with previous studies that examined the effect of adding 2% plant oil in the diets of dairy ewes [33], but was similar to recent reports in dairy cows that found a negative impact of a high level of supplemented oil on DMI [24,34,35,36]. According to Illius et al. [37], voluntary ruminant feed intake is affected by nutrient and energy flows related to ruminal fermentation. Adding high levels of oil in diets that was the case of the current study, may reduce diet acceptability [38], which is caused by ruminal function reduction. Other studies have shown that oil addition to diets reduces fibre digestibility, DMI, and feed palatability in ruminants, suggesting negative effects of plant oils on animals’ appetite. This occurs due to selection against microorganisms with cellulolytic capability, leading to a decrease in ruminal fibre digestion [39]. Moreover, DMI differences among oil supplement groups (with the highest observed in RBO) indicates the effect of oil type on nutrient digestibility [40]. 

Known as an important indicator of cow health status in dairy management, body condition score (BCS) is also regularly used to estimate fatness in the form of energy reserves as well as animal welfare status [41,42,43,44]. A meta-analysis by Kenyon et al. [29] demonstrated a positive association between BCS at breeding and ewe reproductive traits (pregnancy rate and number of lambs born). Generally, these parameters increase as BCS increases from 2.0 to 3.0 [45,46,47]. At the commencement of the feeding trial, the average BCS of the experimental animals was 1.5; a reflection of the low quality pastures the ewes were grazing and a pointer to fat mobilisation from body reserves for sustaining milk synthesis [48]. At the end of the feeding trial, average BCS values of ewes fed CO, RBO, and FSO rose to 2.55, 2.60, and 2.55, respectively. These BCS were within the target of 2.5–3.0 [29], which suggests that the use of such supplements could have a positive effect on not only milk yield, but also reproductive performance and the general welfare of dairy ewes. 

### 4.2. Effect of Dietary Supplements on Milk Yield and Milk Composition

Despite the wide accessibility and availability of canola and rice bran in Australia [49,50], the extent of use of these plant lipid sources as dietary supplements in the Australian dairy industry is unknown. Supplementing diets with canola and rice bran oils in the current study increased milk yield without exerting negative effects on milk fat and protein compositions. Lunsin et al. [24] supplemented dairy cow diets with 2%, 4%, and 6% rice bran oil in a confined system and did not observe any statistical variation in milk production. This was inconsistent with a reduction in the milk yield of dairy goats fed total mixed rations that included 5%, 10%, and 20% rice bran [25]. In contrast, an increase in the milk yield of the RBO group observed in the current study suggests the advanced effect of rice bran oil inclusion in a pasture-based system compared to a confined system. Regarding milk fat and protein concentrations, supplementation of grazing dairy ewes with rice bran oil in the current study, had no influence on milk fat. However, it significantly enhanced milk protein even though the potential to alter milk protein concentration by changing the dietary composition is considered less compared with the potential to alter milk fat composition [9]. This increment of change in protein composition in milk agrees with the findings of Park et al. [25] in goat milk, but disagrees with a decrease observed in cows when the percentage of dietary RBO was increased [24]. On the other hand, supplementation of ewes in this study and cows [19] in similar pasture-based dairy systems with 5% of CO demonstrated an increase in milk yield. However, while inclusion of CO had no statistically significant effect on all milk components of lactating ewes, Otto et al. [19] reported marginal decreases in fat and protein percentages of cow milk. These contrasting results in response to rice bran and canola oil supplementation suggest that there could be physiological differences between species in lipid metabolisms that might need further investigation.

Variations in results assessing the effect of mostly whole or extruded flaxseed [51] and flaxseed oil [52] on milk production and composition of dairy ewes have been reported. Akin to the current results, no statistical difference in milk production was observed when ewes were supplemented with extruded linseed at 128 g/day [53] and 220 g/day [54] or linseed oil at 6% of the estimated total DM intake [52]. These findings were in contrast with other authors who distinguished either an increase [27] or a decrease [28] in milk yield of dairy ewes fed 250 g/day of whole flaxseed or 200 g/day of extruded flaxseed, respectively. Milk fat depression in response to supplementation with FSO in this study was supported by other studies in sheep [53] and cows [34,55,56], but disagrees with others that showed no changes in sheep [27,52,54] or a minor increase in sheep [26,28] and goats [57]. These variations might be due to the multi nutritional effects, including energy balance, NDF concentration, and feed particle size, that have strong correlations with milk yield and milk fat concentration [11]. 

Safflower, which is grown in over 60 countries [58], has been used widely as a supplement in ruminant diets [59]. Despite studies investigating the effects of using various types of safflower on bovine and caprine performance [60], there is relatively little information on its effectiveness as a supplement for influencing milk yield and composition in lactating ewes. In this study, supplementation of grazing dairy ewes with SFO increased milk production by 16%. This supports the findings of Ahmadpour et al. [61], who supplemented dairy cows with rolled safflower seed at 3% and 6% and reported increases in milk yield by 2% and 9%, respectively. Other studies have, however, reported no significant effects on milk yield when the diets of lactating cows [23,59,62,63] and goats [64] were supplemented with safflower oil or seed. Similarly, variable responses and changes in milk components were observed when the diets of lactating goats or cows were supplemented with safflower. Some results portrayed negative effects [23,34,64], which align with our results, while others did not observe any significant effects [59,61,62,63]. The wide range of inclusion rates and variation in dietary components in these studies might have led to the variable responses reported. 

An outstanding enhancement of milk yield by approximately 30% compared to the control animals was observed in ewes supplemented with RPO. Increases in fat (13%) and protein (31%) were also observed. These incremental improvements in milk yield and total solids production play an important role in positively enhancing the economic benefits for dairy sheep producers as most sheep milk is used for cheese making [65]. The quantity of cheese that can be produced from sheep milk is limited by the concentrations of fat and, especially, protein in raw milk [11]. Reviews on bypass fat supplementation studies suggest a consistent increase in the milk production of lactating cows by 5.5%–24% [66], while variable responses were presented in lactating ewes [11]. According to Pulina et al. [11], positive effects of supplementing rumen-protected fat on dairy sheep production performance generally occur with feeding trials longer than 4 weeks. This was confirmed in the current work, while short-term studies had a minor reduction or no change [67,68,69]. In this study, we recorded a reduction in the concentration of milk fat in the RPO group. This agrees with the findings of Rotunno et al. [70], who fed ewes with 4% and 8% rumen-protected fat, whereas this disagreed with the consistent increase in milk fat concentration reported by Pulina et al. [11]. Differences in dietary components, type and dosage of protected fat, feeding regimes, or stage of lactation might have accounted for this contrasting set of outcomes. 

### 4.3. Effect of Breed on Animal Performance

The East Friesian (EF) breed of sheep was developed in northern Germany and the Netherlands, and has become one of the world’s most productive dairy sheep. The EF has earned the reputation as the most productive dairy sheep breed in terms of milk yield [71]. However, it has a low ability to adapt under unfavourable environmental conditions, especially excessive heat and humidity [72]. Thus, this breed has been used widely in crossbreeding systems to improve milk production of local breeds in various temperate environments [72,73,74]. Together with Awassi (AW), the predominant breed in the Eastern Mediterranean countries [75], EF was introduced to Australia in the 1990s, and since, has been used more widely in the dairy sheep industry as reported by the Australian Rural Industries Research and Development Corporation [76]. The improvement in milk yield without any negative effects on the relative content of milk composition in crossbred ewes AW × EF was akin to Clement et al. [77], whereas it was inconsistent with Gootwine and Goot [72], who demonstrated similar milk volumes between AW and AW × EF. Local heat stress that leads to a depression of feed intake, milk production, and reproduction [78,79] might be the principal factor for this performance variation by crossbreds in some studies. Moreover, statistically significant variation in the interaction between treatments and sire breed regarding milk production and composition, but not feed intake, in the current research suggests that gene regulation may be involved in experimental oil metabolism. Therefore, identification of regulated genes for milk yield and composition in response to plant and rumen-protected marine oil supplements needs to be investigated.

## 5. Conclusions

The current study demonstrated that canola, rice bran, safflower, and rumen-protected EPA + DHA could improve lactation traits without any negative impact on BCS of dairy ewes grazing low quality pasture. Under the same nutrition and management conditions, crossbred AW × EF significantly showed greater lactation performance than AW. Utilising these oil supplements combined with crossbreeding the AW and EF sheep breeds is, therefore, recommended for Australian sheep milk producers utilising pasture-based systems. In addition, the novel potential of supplementing dairy sheep with rice bran and canola oils explored in this study may need further research to better elucidate their metabolic mechanisms.

## Figures and Tables

**Table 1:** Ingredient composition of the experimental pellets ^a^.

Items	Control	CO	RBO	FSO	SFO	RPO
Ingredient, g/kg						
Wheat	585	545	535	465	535	530
Paddy rice	210	210	220	280	210	215
Lupins	148	148	148	148	148	148
Canola oil, ml/kg	-	50	-	-	-	-
Flaxseed oil, ml/kg	-	-	-	50	-	-
Safflower oil, ml/kg	-	-	-	-	50	-
Rice bran oil, ml/kg	-	-	50	-	-	-
EPA + DHA, ml/kg	-	-	-	-	-	50
Ammonium sulphate	12.6	12.6	12.6	12.6	12.6	12.6
Salt	10	10	10	10	10	10
Limestone	20.9	20.9	20.9	20.9	20.9	20.9
Sheep premix	1	1	1	1	1	1
Acid buff	6.25	6.25	6.25	6.25	6.25	6.25
Sodium bicarbonate	6.25	6.25	6.25	6.25	6.25	6.25

^a^ Canola oil (CO); rice bran oil (RBO); flaxseed oil (FSO); safflower oil (SFO); rumen-protected oil (RPO).

**Table 2:** Nutrient compositions ^a^ of basal and experimental diets ^b^.

Component (% DM)	Pasture	Hay	Control	CO	RBO	FSO	SFO	RPO
DM	96.5	95.5	91.5	93.0	91.6	90.0	91.7	91.6
OM	90.5	97.3	92.2	93.3	92.7	91.0	91.8	92.0
Ash	9.5	2.7	7.8	6.7	7.3	9.0	8.2	8.0
ADF	45.5	37.6	10.6	7.1	8.1	9.7	9.0	8.5
NDF	69.9	68.3	30.0	21.8	19.4	23.3	23.9	22.0
EE	1.4	1.2	3.3	5.7	5.2	5.4	5.0	5.1
CP	4.7	4.3	14.6	14.0	14.7	14.6	14.5	15.6
TDN	48.5	54.1	73.4	75.9	75.2	74.1	74.5	74.9
ME, MJ/kg DM	7.1	8.1	11.7	12.2	12.0	11.8	11.9	12.0

^a^ Dry matter (DM), organic matter (OM), acid detergent fibre (ADF), neutral detergent fibre (NDF), ether extract (EE), crude protein (CP), total digestible nutrients (TDN) and metabolisable energy (ME); ^b^ Canola oil (CO), rice bran oil (RBO), flaxseed oil (FSO), safflower oil (SFO), rumen-protected oil (RPO).

**Table 3:** Least square means and standard errors (LSM ± SEM) of experimental feed intake ^a^ (g/head/day).

Items	Feed Intake	DMI	OM	ADF	NDF	EE	CP
Treatment ^b^ (T)							
Control	885.5 ^a^	810.3 ^a^	741.4 ^a^	85.9 ^a^	243.1 ^a^	26.7 ^e^	118.3 ^a^
CO	751.3 ^c^	698.7 ^c^	651.9 ^b^	49.6 ^e^	152.3 ^d^	39.8 ^b^	97.8 ^e^
RBO	860.4 ^b^	788.0 ^b^	730.5 ^a^	63.8 ^c^	152.9 ^d^	40.9 ^a^	115.8 ^b^
FSO	754.3 ^c^	678.9 ^d^	617.8 ^d^	65.9 ^b^	158.2 ^c^	36.7 ^c^	99.1 ^e^
SFO	767.1 ^c^	703.4 ^c^	645.8 ^bc^	63.3 ^c^	168.1 ^b^	35.2 ^d^	102.0 ^d^
RPO	753.9 ^c^	690.5 ^cd^	635.3 ^c^	58.7 ^d^	151.9 ^d^	35.2 ^d^	107.7 ^c^
Breed ^c^							
AW	793.5	726.5	678.8	64.3	170.6	35.7	106.5
AW × EF	797.1	729.9	671.9	64.7	171.5	35.8	107.0
SEM	4.1	3.8	3.5	0.6	1.7	0.3	0.6
*p*-values							
Treatment	0.0001	0.0001	0.0001	0.0001	0.0001	0.0001	0.0001
Breed	0.4483	0.4384	0.4423	0.3670	0.3492	0.5652	0.4358
T × Breed	0.7877	0.7982	0.7993	0.7557	0.6935	0.8934	0.8082

^a^ Dry matter intake (DMI), organic matter (OM), acid detergent fibre (ADF), neutral detergent fibre (NDF), ether extract (EE), crude protein (CP); ^b^ Canola oil (CO), rice bran oil (RBO), flaxseed oil (FSO), safflower oil (SFO), rumen-protected oil (RPO); ^c^ Awassi (AW), East Friesian (EF), Awassi × East Friesian (AW × EF) crossbred. Values with different superscripts within columns are significantly different (*p* < 0.05).

**Table 4:** Effect of supplementation with diverse plant-derived oils on body condition score and lactation performance traits ^a^.

Item	MY	FCM	Fat	FY	Protein	PY	Lacto-se	SNF	SCC	BCS
Treatment ^b^ (T)										
Control	484 ^d^	542 ^bc^	7.4 ^a^	36 ^bc^	5.4 ^c^	26 ^c^	4.9	10.9 ^bc^	109 ^a^	2.1 ^c^
CO	525 ^c^	573 ^b^	7.2 ^ab^	38 ^b^	5.5 ^bc^	29 ^b^	4.9	11.1 ^bc^	98 ^ab^	2.3 ^a^
RBO	527 ^c^	578 ^b^	7.2 ^ab^	38 ^b^	5.9 ^a^	31 ^b^	4.9	11.7 ^a^	73 ^c^	2.2 ^bc^
FSO	489 ^d^	523 ^c^	6.9 ^bc^	34 ^c^	5.4 ^c^	26 ^c^	4.8	10.8 ^c^	60 ^c^	2.3 ^a^
SFO	562 ^b^	587 ^b^	6.6 ^c^	37 ^b^	5.6 ^b^	31 ^ab^	4.8	11.2 ^b^	105 ^ab^	2.2 ^bc^
RPO	628 ^a^	649 ^a^	6.6 ^c^	41 ^a^	5.4 ^c^	34 ^a^	4.8	11.0 ^bc^	81 ^bc^	2.2 ^bc^
Breed ^c^ (B)										
AW	496 ^b^	535 ^b^	7.1	35 ^b^	5.5	27 ^b^	4.8 ^b^	11.1	97 ^a^	2.2 ^b^
AW × EF	578 ^a^	617 ^a^	6.9	40 ^a^	5.5	32 ^a^	4.9 ^a^	11.2	78 ^b^	2.3 ^a^
SEM	3.4	7.8	0.07	3.6	0.04	2.9	0.02	0.05	3.6	0.0
*p*-Values										
Treatment	0.0001	0.0001	0.0001	0.0021	0.0001	0.0001	0.1689	0.0001	0.0002	0.0018
Breed (B)	0.0001	0.0001	0.1765	0.0001	0.7444	0.0001	0.0006	0.1351	0.115	0.0030
Week (W)	0.0001	0.0001	0.0001	0.0001	0.0001	0.0001	0.0001	0.0257	0.0012	0.0001
T × B	0.0001	0.0001	0.0001	0.0002	0.0003	0.0001	0.0001	0.0257	0.0795	0.0002
T × W	1.0000	1.0000	0.9766	0.9999	0.8717	1.0000	0.8348	0.8039	0.3630	0.9999
B × W	0.9061	0.8724	0.9494	0.8517	0.9971	0.9380	0.6808	0.9910	0.9974	0.8640

^a^ Milk yield (MY, g/day), fat-corrected milk (FCM, g/day), fat (g/100 g milk), fat yield (FY, g/day), protein (g/100 g milk), protein yield (PY, g/day), lactose (g/100 g milk), solids-non-fat (SNF, g/100 g milk), somatic cell count (SCC, ×1000 cells/mL), body condition score (BCS); ^b^ Canola oil (CO), rice bran oil (RBO), flaxseed oil (FSO), safflower oil (SFO), rumen-protected oil (RPO); ^c^ Awassi (AW), East Friesian (EF), Awassi × East Friesian (AW × EF) crossbred. Values with different superscripts within columns are significantly different (*p* < 0.05).
